# Isolation, *in vitro* culture and identification of a new type of mesenchymal stem cell derived from fetal bovine lung tissues

**DOI:** 10.3892/mmr.2015.3854

**Published:** 2015-05-27

**Authors:** PENGFEI HU, YABIN PU, XIAYUN LI, ZHIQIANG ZHU, YUHUA ZHAO, WEIJUN GUAN, YUEHUI MA

**Affiliations:** 1 Institute of Animal Sciences, Chinese Academy of Agricultural Sciences, Beijing 100193, P.R. China; 2 Institute of Special Economic Animal and Plant Sciences, Chinese Academy of Agricultural Sciences, Changchun, Jilin 130112, P.R. China; 3 Harbin Institute of Physical Education, Harbin, Heilongjiang 150008, P.R. China

**Keywords:** lung-derived mesenchymal stem cells, *in vitro* culture, biological characteristics, fetal bovine

## Abstract

Lung-derived mesenchymal stem cells (LMSCs) are considered to be important in lung tissue repair and regenerative processes. However, the biological characteristics and differentiation potential of LMSCs remain to be elucidated. In the present study, fetal lung-derived mesenchymal stem cells (FLMSCs) were isolated from fetal bovine lung tissues by collagenase digestion. The *in vitro* culture conditions were optimized and stabilized and the self-renewal ability and differentiation potential were evaluated. The results demonstrated that the FLMSCs were morphologically consistent with fibroblasts, were able to be cultured and passaged for at least 33 passages and the cell morphology and proliferative ability were stable during the first 10 passages. In addition, FLMSCs were found to express CD29, CD44, CD73 and CD166, however, they did not express hematopoietic cell specific markers, including CD34, CD45 and BOLA-DRα. The growth kinetics of FLMSCs consisted of a lag phase, a logarithmic phase and a plateau phase, and as the passages increased, the proliferative ability of cells gradually decreased. The majority of FLMSCs were in G0/G1 phase. Following osteogenic induction, FLMSCs were positive for the expression of osteopontin and collagen type I α2. Following neurogenic differentiation, the cells were morphologically consistent with neuronal cells and positive for microtubule-associated protein 2 and nestin expression. It was concluded that the isolated FLMSCs exhibited typical characteristics of mesenchymal stem cells and that the culture conditions were suitable for their proliferation and the maintenance of stemness. The present study illustrated the potential application of lung tissue as an adult stem cell source for regenerative therapies.

## Introduction

Respiratory disease is one of the most common and severe diseases in the world. The occurrence of respiratory diseases is generally caused by an imbalance of lung stem cell differentiation and the loss of functional cells. As with bone marrow, fat and other tissues in the body, the lung also contains a group of cells, termed lung-derived mesenchymal stem cells (LMSCs), which possess the capacity for self-renewal and potential for multilineage differentiation. LMSCs can differentiate into numerous cell types under specific conditions, including mesoderm and neural ectoderm-derived cells (1). A previous study investigating the biological characteristics of LMSCs illustrated the role of LMSCs in lung tissue repair and regenerative processes (2). While the lung consists of >40 types of cells derived from all three embryonic germ layers (3), the internal structure is complicated. Furthermore, due to a lack of specific lung stem cell surface markers, they have rarely been investigated, and their existence and source have often been disputed. Since the discovery of labeling proliferative epithelial cells with tritium-labeled thymine or 5-bromo-2-deoxyuridine, the existence of lung stem cells has now been confirmed. Certain cell types with stem cell properties have been found in basal cells (4), Clara cells (5) and alveolar epithelial cells type II (6). However, LMSCs are scarce and do not possess distinctive phenotypic characteristics, thus preventing further investigation of LMSCs.

In certain studies investigating LMSCs in humans, mice and rats, the mesenchymal phenotype and differentiation ability of cultured human adult bronchial fibroblast-like cells have been analyzed and compared with those of mesenchymal cell progenitors isolated from fetal lung and adult bone marrow tissues (1,7). It was illustrated that they were able to differentiate along the three adipogenic, osteogenic and chondrogenic mesenchymal pathways (1). The results indicated that MSCs are present in human adult lung and may be actively involved in lung tissue repair under physiological and pathological conditions (1). These characteristics indicate that LMSCs could be used as powerful tools in reconstructive medicine, however, certain problems remain unresolved, for example, the origin, immunoregulation, purification and lack of specific surface markers.

In the present study, bovine FLMSCs were isolated and induced to differentiate into osteoblasts and nerve cells, thereby establishing a larger experimental animal model for this type of research. Furthermore, the present study further examined these cells, including analysis of their growth kinetics, colony-forming rate and the cell cycle as well as detection of a special surface antigen, in order to identify the biological characteristics of this cell line. There are numerous advantages in using a large animal experimental model in this type of research, particularly prior to the transfer of regenerative technology to human medicine, as large animals are biomechanically more similar to humans than small experimental animals (8–10).

## Materials and methods

### Isolation, culture and purification of bovine FLMSCs

All procedures involving animals were approved by the animal care and use committee at the Institute of Animal Sciences, Chinese Academy of Agricultural Sciences (Beijing, China) where the experiment was conducted. All procedures involving animals were approved and authorized by the Chinese Ministry of Agriculture (Beijing, China).

Lung tissues were separated from 3–5 month-old bovine fetuses and the trachea and bronchus were removed from the lung under aseptic conditions. The tissues were washed three times with phosphate-buffered saline (PBS) containing 104 IU/ml penicillin/streptomycin to remove connective tissue membranes and capillaries. The tissues were cut into 1 mm^3^ sections and digested with 0.2% (m/v) type II collagenase (Sigma-Aldrich, St. Louis, MO, USA) at 37°C for 30 min. Enzymatic digestion was then neutralized with α-modified Eagle's medium (α-MEM; Gibco-BRL, Carlsbad, CA, USA) supplemented with 10% (v/v) fetal bovine serum (FBS; Biochrom, Holliston, MA, USA). The suspension was filtered with a 74-*µ*m-mesh sieve and centrifuged at 300 × g for 10 min at room temperature. The supernatant was discarded and the pellet was resuspended in complete medium containing α-MEM, 10% (v/v) FBS and 104 IU/ml penicillin/streptomycin. The cell suspension was plated and incubated at 37°C with 5% CO_2_. At 24 h after initial plating, the cells were washed twice with PBS to remove non-adherent cells.

When the cells reached 70–80% confluence, 0.25% (m/v) trypsin (Gibco-BRL) was added to dissociate the cells from the plates, and then trypsinization was terminated with complete medium. Cells were subcultured into new plates and incubated at 37°C with 5% CO_2_. Generally, following three to four passages, the cells were purified.

The bovine FLMSCs in logarithmic phase were enumerated under a hemocytometer, pelleted and resuspended in freezing medium [10% dimethyl sulfoxide (DMSO), 40% FBS and 50% α-MEM] at a concentration of 3×10^6^ viable cells per milliliter. Cell suspension was aliquoted into sterile plastic cryovials labeled with the species, freezing serial number and date. The vials were sealed and kept at 4°C for 20–30 min to equilibrate the DMSO, placed into a programmed cryopreservation system and then transferred to liquid nitrogen for long-term storage. Tubes taken from the liquid nitrogen were thawed in a 42°C water bath, then transferred to flasks with α-MEM containing 10% FBS and finally cultured at 37°C with 5% CO_2_. The medium was renewed after 24 h.

### Surface marker detection

Cells of passages 3, 9, 17 and 25 were fixed in 4% (m/v) paraformaldehyde for 15 min and then washed with PBS three times (5 min each). The cells were permeabilized using 0.2% (v/v) Triton X-100 for 20 min and washed three times (5 min each) with PBS. Cells were blocked by 10% (v/v) goat serum (Beijing Zhongshan Golden Bridge Biotechnology Co., Ltd., Beijing, China) for 30 min and then incubated in 3% (m/v) bovine serum albumin containing the following antibodies: i) Rabbit polyclonal anti-bovine CD29 (1:100; cat. no. ab115146; Abcam, Cambridge, MA, USA); ii) rabbit polyclonal anti-bovine CD44 (1:100; cat. no. ab157107; Abcam) for 1 h at room temperature. The samples were washed three times (each 10 min) with PBS and then the cells were incubated in PBS containing fluorescein isothiocyanate-conjugated goat anti-rabbit secondary antibody (1:500; Beijing Zhongshan Golden Bridge Biotechnology Co., Ltd.; cat. no. ZF-0511) for 1 h at 37°C. Following incubation, the cells were washed three times with PBS (each 10 min). Finally, the cells were counterstained with DAPI (Sigma-Aldrich) and examined using a Nikon TE-2000-E confocal microscope (Nikon, Tokyo, Japan).

### Reverse transcription polymerase chain reaction (RT-PCR) analysis

RNA was extracted from cells of passage 3, 9, 17 and 25 using TRIzol reagent (Invitrogen Life Technologies, Carlsbad, CA, USA). Template cDNA was prepared using a reverse transcription system (Takara Bio, Inc., Shiga, Japan) and then amplified by PCR using the specific primers listed in Table I. The PCR products were visualized by 2% agarose gel electrophoresis.

### Growth kinetics

Cells of passages 3, 9, 17 and 25 were used to analyze the growth kinetics of bovine FLMSCs. The cells were harvested and plated in 24-well microplates at a density of 1×10^4^ cells/well. The cells from three random wells were counted each day for 8 days. Growth curves were plotted according to the mean values and the population doubling time was calculated using the growth curve.

### Colony-forming assay

Cells of passages 2, 8, 16, 24 were seeded in a 60 mm culture-plate at a density of 50 cells/well and cultured for 7–10 days, and the number of colony-forming units was counted to calculate the colony-forming rate, which was calculated using the following formula: (Colony-forming unit numbers / starting cell number) ×100%.

### Cell cycle analysis

Cells of passages 3, 9, 17 and 25 were harvested and centrifuged at 200 × g at room temperature for 5 min. Following discarding the supernatant, the pellet was washed three times with PBS at pH 7.4 and then resuspended in precooled 70% (v/v) ethanol overnight at 4°C. The samples were centrifuged at 200 × g for 5 min to remove the ethanol, washed once with PBS at pH 7.4, resuspended in propidium iodide (PI) solution (0.05 mg/ml PI, 0.02 mg/ml RNase, 0.585 g/ml NaCl, 1 mg/ml sodium citrate, pH 7.2–7.6) and incubated at 4°C for 30 min in the dark. Samples were then analyzed by flow cytometry.

### Osteogenic differentiation

The cells were divided into two groups: The induced group and the control group. When cultures reached 50–60% confluence, cells of the induced group were incubated in osteogenic medium containing 0.1 mM dexamethasone (Sigma-Aldrich), 10 mM β-glycerophosphate (Sigma-Aldrich) and 50 mg/l vitamin C. In addition, cells of the control group were cultured in complete medium without any inducer. The medium was replaced every 2 days. The cells were cultured for 2 weeks until significant calcium deposits were observed. The cells were then washed and fixed using 4% paraformaldehyde solution and stained using Alizarin Red S (Sigma-Aldrich), which specifically stains calcified deposits in the extracellular matrix. Additionally, the expression of osteoblast-specific genes, osteopontin (OPN) and taurus collagen type I α2 (COL1A2), was analyzed by RT-PCR.

### Neurogenic differentiation

The cells were divided into two groups: The induced group and the control group. When cultures reached 50–60% confluence, cells of the induced group were used for two rounds of induction, primarily, cells were cultured in the pre-induced media consisting of low glucose-DMEM, 10% FBS and 1 mM β-mercaptoethanol for 24 h. The induced media consisted of low glucose-DMEM, 10% FBS, 200 *µ*M butylated hydroxyanisole (BHA) and 2% DMSO, which was added for 5 days. The induced cells were collected to identify the expression of microtubule-associated protein 2 (MAP-2) and nestin by RT-PCR.

## Results

### Isolation, culture and morphology of bovine FLMSCs

**Figure 1:**
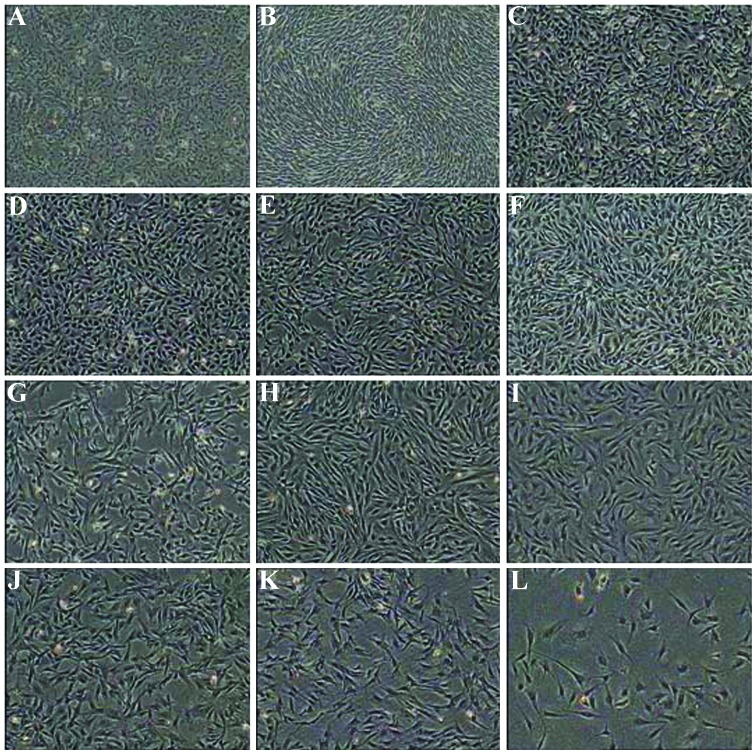
Morphology of bovine FLMSCs *in vitro*. (A) P0 FLMSCs prior to passage; (B) P2 FLMSCs prior to passage; (C) P3 FLMSCs prior to passage; (D) P8 FLMSCs prior to passage; (E) P9 FLMSCs prior to passage; (F) P11 FLMSCs prior to passage; (G) P14 FLMSCs prior to passage; (H) P17 FLMSCs prior topassage; (I) P22 FLMSCs prior to passage; (J) P25 FLMSCs prior to passage; (K) P30 FLMSCs prior to passage; (L) P33 FLMSCs prior to passage. FLMSCs, fetal lung-derived mesenchymal stem cells.

In primary culture, the cells expanded easily and exhibited a fibroblast-like morphology. Numerous bovine FLMSCs were mixed with epithelioid cells (Fig. 1A), however, following three to four passages, the epithelioid cells were detached and eliminated from the population (Fig. 1B and C). The FLMSCs were arranged in a whirl-like pattern (Fig. 1D–F). No clear morphological differences were identified among different passages and the biocharacteristics were stable following serial passage (Fig. 1G–K). The cells were cultured to passage 33 and exhibited a representative senescent appearance, including blebbing and karyopyknosis in the majority of cells (Fig. 1L). Eventually, as the passage number increased, the cells detached from the plates.

### Identification of bovine FLMSCs

**Figure 2:**
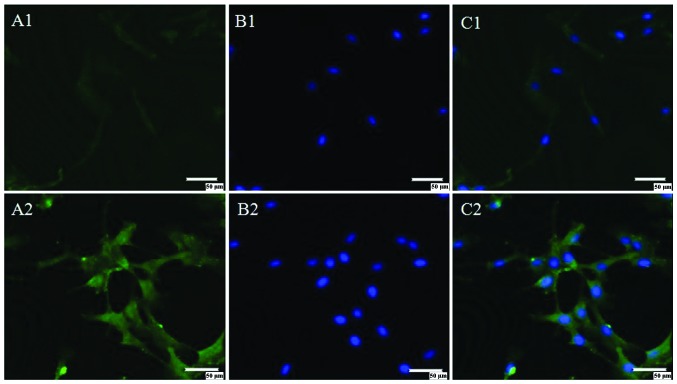
Specific surface markers of bovine fetal lung-derived mesenchymal stem cells from passages 9 detected by immunofluorescence. (A1) CD29; (A2) CD44; (B1 and B2) 4′,6-diamidino-2-phenylindole; (C1 and C2) merged.

**Figure 3:**
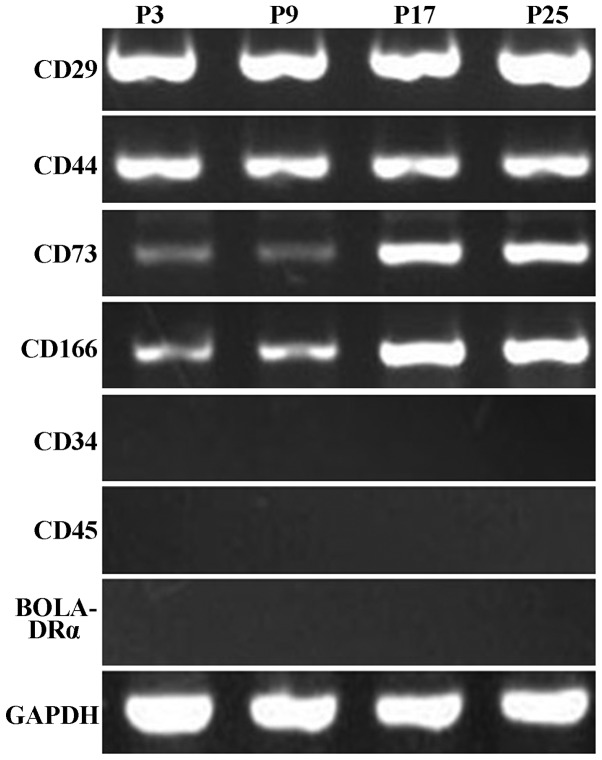
Specific surface markers of bovine fetal lung-derived mesenchymal stem cells from passages 3, 9, 17 and 25 detected by reverse transcription-polymerase chain reaction. BOLA, bovine lymphocyte Ag.

The specific surface antigen markers of bovine FLMSCs from passages 3, 9, 17 and 25 were detected via immunofluorescence and RT-PCR assays. The results of immunofluorescence staining demonstrated that the LMSCs were CD29 and CD44 positive (Fig. 2). RT-PCR indicated that the FLMSCs expressed CD29, CD44, CD73 and CD166. However, the expression of CD34, CD45 and BOLA-DRα was negative (Fig. 3).

### Growth kinetics of bovine FLMSCs

**Figure 4:**
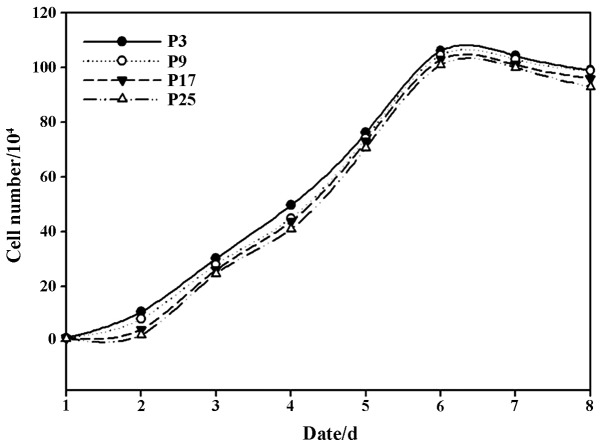
Growth curves of bovine FLMSCs. FLMSCs entered logarithmic phase after day 2, followed by a plateau phase after 6 days and cell growth eventually decreased 7 days later. FLMSCs, fetal lung-derived mesenchymal stem cells.

The growth kinetics of bovine FLMSCs from different passages are shown in the growth curves, which were all typically sigmoidal. The FLMSCs entered logarithmic phase after ~day 2, followed by a plateau phase after 6 days and cell growth eventually decreased ~7 days later (Fig. 4). The average population doubling time of the FLMSCs was 25 h.

### Colony-forming cell assay

**Figure 5:**
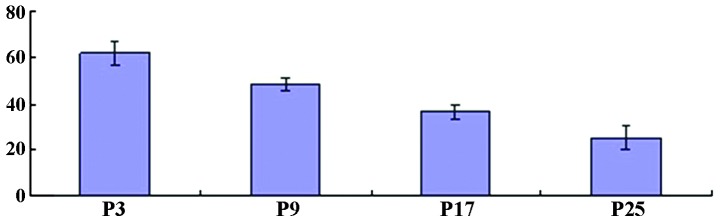
Cloning efficiency of passages 3, 9, 17 and 25 in FLMSCs. The colony-forming rates were 62.01±5.29, 48.66±3.06, 36.67±3.06 and 25.33±5.03% for passage 3, 9, 17 and 25, respectively, demonstrating the self-renewal ability of the cultured bovine FLMSCs. FLMSCs, fetal lung-derived mesenchymal stem cells.

Colony formation was observed under the microscope after 7 days. The colony-forming rates were 62.01±5.29, 48.66±3.06, 36.67±3.06 and 25.33±5.03% for passage 3, passage 9, passage 17 and passage 25, respectively, demonstrating the self-renewal ability of the cultured bovine FLMSCs (Fig. 5).

### Cell cycle analysis

**Figure 6:**
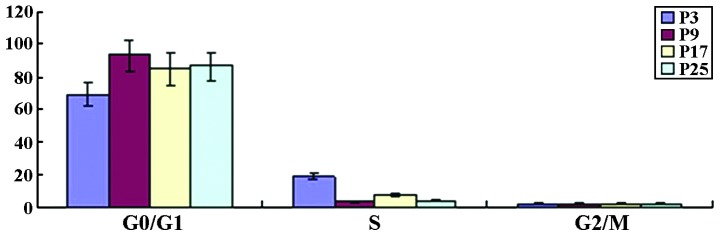
Analysis of DNA content in different passages. No significant difference was identified among passages 3, 9, 17 and 25 in different phases of the cell cycle.

The cell cycle distribution of cells from passages 3, 9, 17 and 25 is shown in Fig. 4. Flow cytometric analysis demonstrated that the majority of cells of these four passages were in the G0/G1 phase and a small proportion were proliferating cells (in S phase). It was generally considered that this type of phenomenon was correlated with cell cycle progression of MSCs. The cell proportion of different cell phases was analyzed and no significant difference was identified among passages 3, 9, 17 and 25 in different cell phases (P>0.05; Fig. 6).

### Osteogenic differentiation of bovine FLMSCs

**Figure 7:**
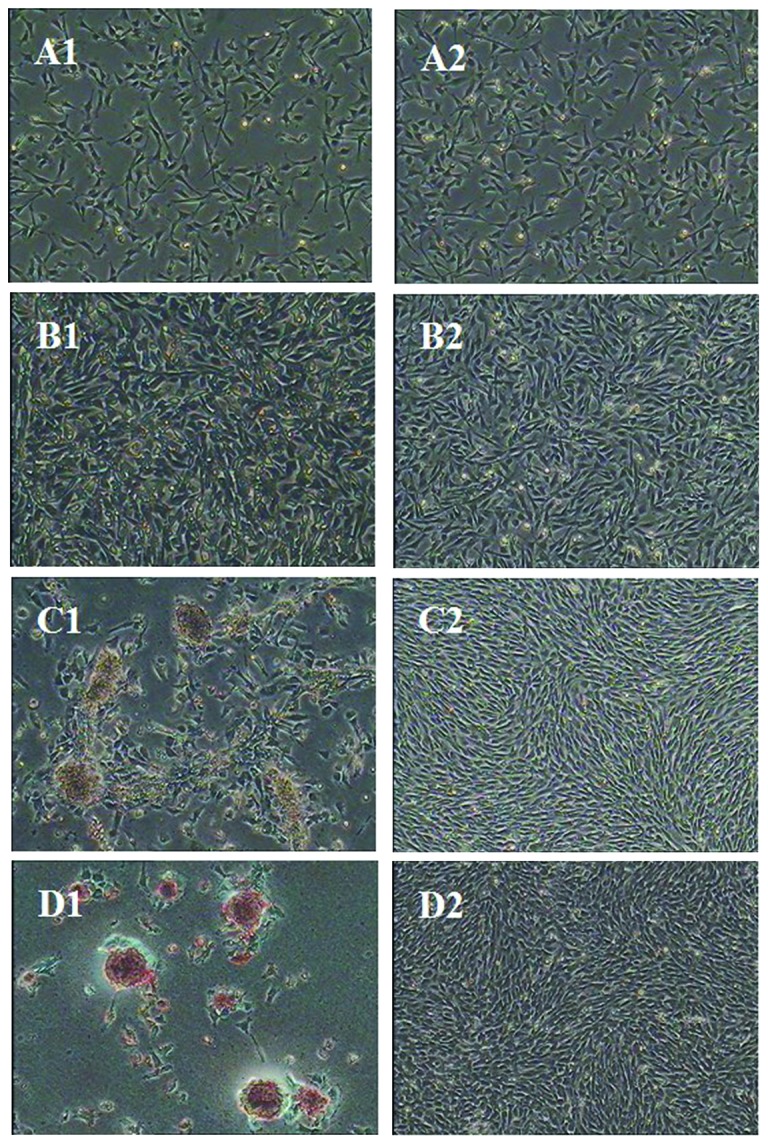
Cellular morphology and Alizarin red staining of osteogenic differentiation. (A1–D1) Cellular morphology following induction for 0, 3 and 7 days and Alizarin red staining. (A2–D2) Cellular morphology following 0, 3 and 7 days of culture and Alizarin red staining in the control group.

Following incubation in osteogenic medium for 7 days, morphological alterations in bovine FLMSCs were visible. The cells altered from a fusiform to tridimensional shape and then aggregated and formed mineralized nodules with increasing culture time (Fig. 7A1 and B1). Furthermore, the nodules were Alizarin Red positive (Fig. 7C1). In addition, as a result of the continuing effects of the inducers, the nodules increased and grew in size (Fig. 7D1). As for the control group, the cells cultured in complete medium were not morphologically altered or stained by Alizarin Red (Fig. 7A2–D2).

**Figure 8:**
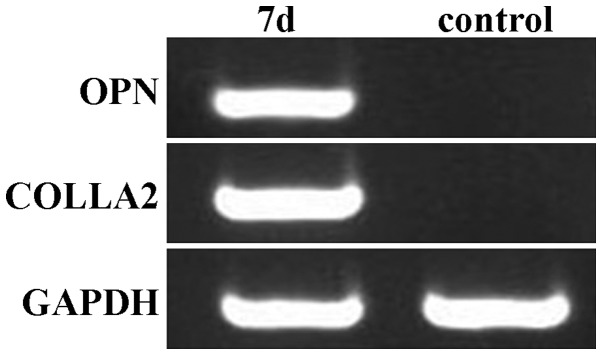
Reverse transcription polymerase chain reaction of osteoblast-specific genes. OPN and COLLA2 were expressed in the induced group following incubation for 7 days, however, these genes were not expressed in the control group. OPN, osteopontin; COLLA2, collagen type I α2.

Osteogenic differentiation of bovine FLMSCs was analyzed by RT-PCR. The specific genes, including OPN and COLLA2, were detected in the induced group, whereas the expression of these genes was not detected in the control group (Fig. 8).

### Neurogenic differentiation of bovine FLMSCs

**Figure 9:**
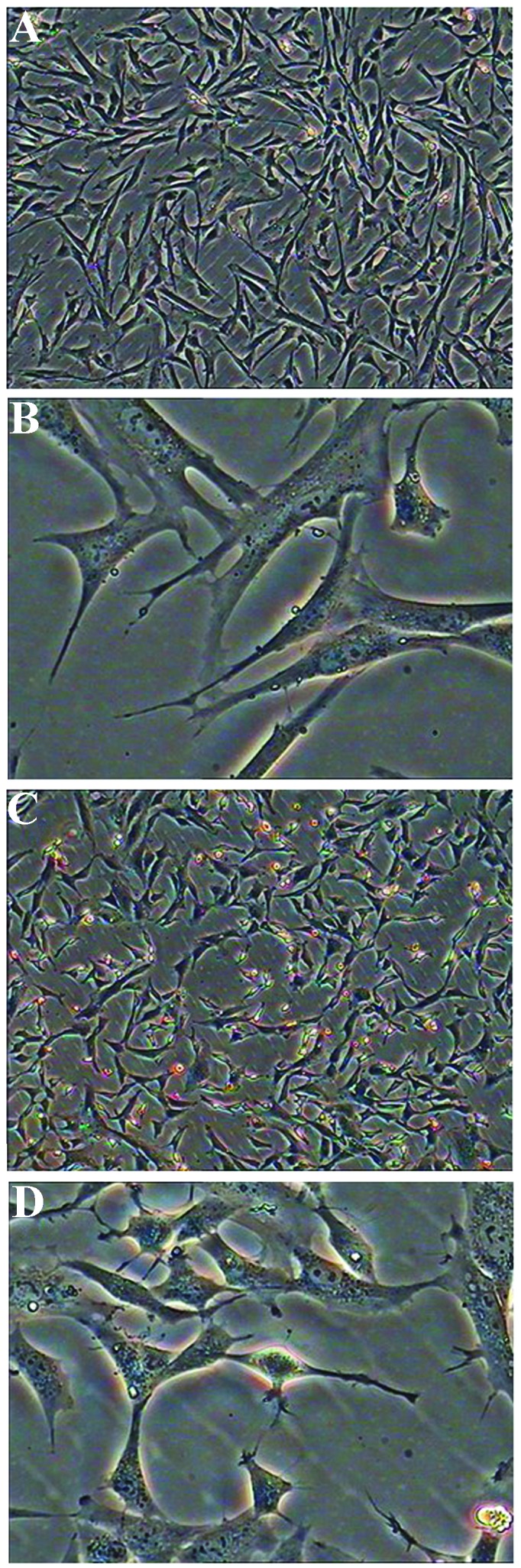
Morphological alterations of neurogenic differentiation. (A) Prior to induction (magnification, ×40); (B) prior to induction (magnification, ×200); (C) following induction (magnification, ×40); (D) following induction (magnification, ×200).

**Figure 10:**
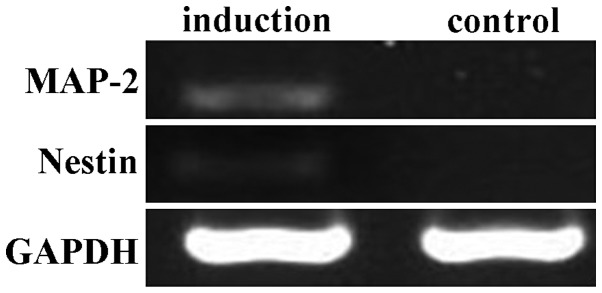
Reverse transcription polymerase chain reaction of neurocyte-specific genes. MAP-2 and nestin were expressed in the induced group following incubation, however, these genes were not expressed in the control group. MAP-2, microtubule-associated protein 2.

Bovine FLMSCs cultured in the pre-induced media grew actively and the morphology of induced cells was not altered (Fig. 9). Following addition of the induced media for 30 min, cell contraction appeared at the cell body and a long cell process formed around the cells. Approximately 50% of the cells demonstrated a typical unipolar, bipolar and multipolar neuron-like cell morphology following addition of the induced media for 4 h. Positive MAP-2 and nestin expression, which are marker genes of neurons, was detected by RT-PCR (Fig. 10). The results indicated that bovine FLMSCs can differentiate into ectoderm cell types.

## Discussion

Numerous factors, including insufficient genetic information and progress compared with other fields, unawareness of its therapeutic potentials, and an unwillingness to investigate animals other than model species are all responsible for the lack of research on bovine FLMSCs compared with those from other mammals. In the present study, bovine FLMSCs were successfully isolated from 3–5 month old fetal bovine lung tissues. The FLMSCs could be cultured and passaged *in vitro* for at least 33 passages and could be induced to differentiate into different cell types. Compared with mammal MSCs, bovine FLMSCs exhibited a good proliferative ability *in vitro* and proliferation appeared to decrease from low to high passage. This phenomenon matched the animal cytophysiology *in vitro*, however, the cells demonstrated a good proliferative ability when the culture condition was improved. Bovine FLMSCs could be preliminary purified by serial passage to remove epithelial cells but not fibroblasts. To further purify the bovine FLMSC population, fluorescence activated cell sorting or magnetic sorting could be performed.

The colony-forming ability is essential for the self-renewal and specific differentiation characteristics of stem cells. Not every adhered cells can proliferate and form colonies. The colony-forming ability can reflect two important traits of the cell, namely population-dependent and proliferative capacity. The colony-forming rate is directly associated with the seeding density of cells, thus, the seeding cells must be dispersed into a single cell suspension. In the present study, the result of the colony-forming assay demonstrated that the colony-forming rate was between 36.67 and 62.01% at passages 3, 9 and 17, and the rate was significantly decreased to 25.33 at passage 25, indicating that the cultured bovine FLMSCs had good self-renewal and proliferative ability. The result was in accordance with the growth kinetics analyzed in the present study.

To date, specific markers of bovine FLMSCs have not been found, thus the surface markers of mesenchymal stem cells, including CD29, CD44, CD73 and CD166 were detected to identify the surface antigen. As a control, the specific markers of hematopoietic stem cell surface antigens CD34, CD45 and BOLA-DRα were detected to eliminate false positive cells (11–13). CD29 is an integrin subunit associated with late-stage antigen receptors. CD29 is essential for cell adhesion and recognition in a variety of processes, including embryogenesis, hemostasis, tissue repair, the immune response and metastasis of malignant cells (14). CD44 contributes to a cell-surface glycoprotein involved in cell-cell interactions, cell adhesion and migration. This protein is involved in a wide variety of cellular activities, including lymphocyte activation, recirculation and homing, hematopoiesis and metastasis (15). CD73, also known as ecto-50-nucleotidase, is used as a marker for lymphocyte differentiation. In the present study, the results of immunofluorescence staining demonstrated that bovine LMSCs were CD29 and CD44 positive. The results from RT-PCR indicated that bovine FLMSCs expressed CD29, CD44, CD73 and CD166, whereas CD34, CD45 and BOLA-DRα were not expressed. These results suggested that bovine FLMSCs are a group of mesenchymal stem cells different from hematopoietic stem cells.

An important feature of stem cells is a period of dormancy, the stem cells can only maintain their self-renewal and differentiation capacity in a dormant state. MSCs remain in a resting stage *in vivo*, however, a small number of cells are in an active proliferative phase *in vitro*. A previous study on the stem cell cycle demonstrated that ~10% of MSCs are in the S and G2/M phase, which is the active proliferative phase of stem cells and ~90% of the entire cells are in the G0/G1 phase (16). This phenomenon indicates that MSCs have a high differentiation potential. In the present study, the cell cycle of bovine FLMSCs was assessed by flow cytometry. The percentage of cells in the S phase in passage 3 was 25.03%, indicating that the cells had a strong proliferative capacity when the cells were no longer in an *in vivo* environment. With increasing passage, the cells in the G0/G1 phase increased and the S phase cells decreased, indicating that the proliferative capacity of cells gradually decreased.

The multipotency of stem cells is one of the most important prerequisites for autologous cell therapy (17,18). It was found that mouse BMSCs from different strains have different differentiation abilities, however, the majority of MSCs can differentiate into a variety of cells, including adipocytes (19), osteoblasts (20), chondrocytes (21–23), muscle cells (24), nerve cells (25,26), liver cells (27) and pancreatic β cells (28) under particular inducing conditions. In the present study, bovine FLMSCs were induced to differentiate into osteoblasts and nerve cells and associated genes of these cell types were detected. The results demonstrated that different inducing factors could affect the direction of differentiation of bovine FLMSCs. The induction effect of dexamethasone depended on its concentration (29). Dexamethasone promoted bovine FLMSCs to differentiate into osteoblasts at a range of 10^−8^–10^−10^ mol/l, while it inhibited the differentiation of bovine FLMSCs into osteoblasts and induced bovine FLMSCs to differentiate into lipocytes at a higher concentration (30). However, the detailed mechanisms underlying the effect of dexamethasone on bone formation remain to be elucidated.

Since the first study demonstrating that MSCs are able to differentiate into nerve cells (31), two induction patterns, including a growth/trophic factor-based pattern and organic mutagens pattern (32), have been established. In the present study, a combination of BHA, DMSO and β-mercaptoethanol led to the differentiation of bovine FLMSCs into neurocytes. In addition, the expression level of MAP-2 and nestin was assessed and the results demonstrated that a positive expression of MAP-2 and nestin was detected following induction. The results indicated that bovine FLMSCs could differentiate into ectoderm cell types.

The autologous nature of bovine FLMSCs, together with their putative multipotentiality and convenient procurement, renders them an excellent option for future tissue-engineering and cell-based therapies (33). Although the multilineage differentiation of bovine FLMSCs was successful *in vitro*, there are a number of technical concerns for utilizing these cells in tissue recovery *in vivo*, including a high decrease and an unstable phenotype. These aspects must be taken into consideration in future studies and applications.

## Figures and Tables

**Table I:** Primer sequences used in reverse transcription polymerase chain reaction.

Gene	Primer sequence	Tm (°C)	Fragment size (bp)	Cycle
CD29	F: 5′-GAAACTTGGTGGCATCGT-3′			
	R: 5′-CTCAGTGAAGCCCAGAGG-3′	60	493	30
CD44	F: 5′-CGGAACATAGGGTTTGAGA-3′			
	R: 5′-GGTTGATGTCTTCTGGGTTA-3′	56	301	30
CD73	F: 5′-CAATGGCACGATTACCTG-3′			
	R: 5′-GACCTTCAACTGCTGGATA-3′	60	426	30
CD166	F: 5′-TATCAGGATGCTGGAAAC-3′			
	R: 5′-TAGCCAATAGACGACACC-3′	55	498	30
CD34	F: 5′-CCTCATCAGCTTTGCGACTT-3′			
	R: 5′-CCAGGAGCAAGGAGCACA-3′	60	454	30
CD45	F: 5′-CTACCCAACCTTCTACTCAA-3′			
	R: 5′-TTCACATCCAGGAGGTTC-3′	56	221	30
BOLA-DRα	F: 5′-AGCACTTGGGTTTGAATG-3′			
	R: 5′-GGAGCAGTAGACAGGGAAT-3′	58	404	30
COLLA2	F: 5′-AGAAGCATGTCTGGGTAGGAG-3′			
	R: 5′-AGGATAGGCAGGCGAGATR-3′	59	358	30
OPN	F: 5′-ATACCCTCCCAAGTAAGTC-3′			
	R: 5′-TGTGATGTGAAGTCCTCC-3′	56	340	30
MAP-2	F: 5′-GAGAACGGAATCAACGGAGAAC-3′			
	R: 5′-CCAAACAGAGTGGGAGGTGC-3′	60	396	30
Nestin	F: 5′-AGAGGAGAACGCTGAGTCATT-3′			
	R: 5′-TCTGTAGGCTTTAGTGGTTCTG-3′	54	537	30
GAPDH	F: 5′-GGCAAGTTCAACGGCACAGTCA-3′			
	R: 5′-TAAGTCCCTCCACGATGCCAAAG-3′	60	365	30

Tm, temperature, F, forward; R, reverse; MAP-2, microtubule-associated protein 2; OPN, osteopontin; COLLA2, collagen type I α2; BOLA, bovine lymphocyte Ag.
